# Anti-Obesity, Lipid-Lowering, and Anti-Hyperglycemic Effects of CB-02 in High-Fat-Diet-Induced Obese Mice

**DOI:** 10.3390/molecules30183678

**Published:** 2025-09-10

**Authors:** Hoang Lan Hiep, Phung Van Bang, Nguyen Dinh Nhan, Nguyen Hoang Ngan, Dao Cuong To, Nguyen Van Dung, Le Hong Phu

**Affiliations:** 1 Military Institute of Traditional Medicine, 442 Kim Giang, Dinh Cong, Hanoi 11717, Vietnam; hoanghiep.yhct@gmail.com (H.L.H.); phungbangbs8@gmail.com (P.V.B.); nhantom@yahoo.com (N.D.N.); 2 Department of Pharmacology, Institute of Pharmacy Education, Vietnam Military Medical University, 160 Phung Hung, Ha Dong, Hanoi 12108, Vietnam; nganvmu@gmail.com; 3 Phenikaa University Nano Institute (PHENA), Phenikaa School of Engineering (PSE), Phenikaa University, Nguyen Van Trac, Duong Noi, Hanoi 12116, Vietnam; cuong.todao@phenikaa-uni.edu.vn; 4 Department of Pharmacology, Thai Nguyen University of Medicine and Pharmacy, 284 Luong Ngoc Quyen, Phan Dinh Phung, Thai Nguyen 24124, Vietnam; dung.dhdtn@gmail.com

**Keywords:** CB-02 capsules, *Phyllanthus emblica*, *Dendrobium catenatum*, *Gynostemma pentaphyllum*, obesity, dyslipidemia

## Abstract

Obesity, along with dyslipidemia and hyperglycemia, is a metabolic disorder growing in prevalence that is linked to chronic diseases such as atherosclerosis, hypertension, and type 2 diabetes. This study evaluated the anti-obesity, lipid-lowering, and anti-hyperglycemic effects of CB-02 capsules containing dry extracts of *Phyllanthus emblica* L., *Dendrobium catenatum* Lindl., and *Gynostemma pentaphyllum* in HFD-induced obese Swiss albino mice. After 12 weeks of HFD induction, mice were treated orally with CB-02 (576 or 1152 mg/kg/day) for 8 weeks. CB-02 significantly reduced BW gain, AC, the Lee obesity index, and the relative weights of visceral fat and major organs. It also improved lipid profiles by decreasing TC, TG, LDL-C, and non-HDL-C, while increasing HDL-C. These effects were comparable to orlistat (60 mg/kg/day). Furthermore, CB-02 lowered fasting glucose and improved insulin sensitivity, as indicated by an increased QUICKI and HOMA-β and reduced HOMA-IR. Histopathological evaluation showed that CB-02 reduced hepatic steatosis and inflammatory cell infiltration and also attenuated β-cell morphological alterations and pancreatic histopathological damage. These results suggest that CB-02 may be a promising therapeutic candidate for managing obesity and its comorbidities, including dyslipidemia, hyperglycemia, and hepatic steatosis, contributing to the prevention of type 2 diabetes and cardiovascular diseases in obese individuals.

## 1. Introduction

Obesity, dyslipidemia, and hyperglycemia are three common metabolic disorders that are rapidly increasing in prevalence worldwide. They have become major risk factors for chronic conditions such as atherosclerosis, hypertension, coronary artery disease, type 2 diabetes, and metabolic syndrome [1,2]. The primary causes of these disorders include imbalances in lipid and energy metabolism, often resulting from high-fat diets, sedentary lifestyles, and environmental or genetic factors [3,4,5].

In addition to modern treatment approaches, such as lipid-lowering agents and anti-obesity medications, there is a growing interest in the use of natural herbal preparations due to their safety and minimal side effects [6,7]. In Vietnam’s rich pharmacopeia, many medicinal plants traditionally used for lipid metabolism regulation and weight management have shown potential through both folk medicine and modern research [8,9,10].

*Phyllanthus emblica* (also known as Indian gooseberry) is rich in polyphenols and vitamin C and has been reported to possess strong antioxidant, hypolipidemic, and hepatoprotective properties. Hsu et al. (2025) demonstrated that polysaccharides extracted from *P. emblica* improved lipid metabolism and reduced obesity in HFD-fed mice by enhancing lipolysis via adipose triglyceride lipase, HSL, AMPKα, and β-oxidation, such as PPARα and CPT-1, and reducing lipogenesis, while also modulating the gut microbiota [11]. In a randomized double-blind clinical trial, Usharani et al. (2019) found that administering *P. emblica* extract (500 mg, twice daily) to patients with metabolic syndrome significantly reduced TC (11.1%), LDL-C (21.8%), and TG (19.2%), while increasing HDL-C [12].

*Gynostemma pentaphyllum*, a plant rich in gypenosides with structures similar to ginsenosides, has been shown to regulate lipid metabolism, promote weight loss, and support the treatment of metabolic syndrome. A preclinical study on C57BL/6N mice revealed that *G. pentaphyllum* extract reduced BW, fat mass, cholesterol, and triglycerides by activating AMPK and increasing SIRT1, CPT-1, and HSL expression, while downregulating adipogenesis-related genes such as CCAAT/enhancer-binding protein alpha, PPARγ, and SREBP-1c [13]. Another study confirmed the anti-obesity and hypolipidemic effects of gypenosides in obese mice through lipid metabolism modulation and lipidomics analysis [14].

*Dendrobium catenatum* Lindl. (synonym *Dendrobium officinale* Kimura et Migo), a valuable herb in traditional medicine known for its yin-nourishing and fluid-generating properties, also exhibits effects on energy metabolism and anti-inflammation. Polysaccharides from *D. catenatum* have been shown to improve insulin resistance and lipid metabolism disorders in both experimental mouse models and in vitro studies. *D. catenatum* reduced TG, TC, LDL-C, and FFA levels, while increasing HDL-C, partly through its effects on PPARγ activation [15]. Zhou et al. (2025) demonstrated that *D. catenatum* extract alleviated hepatic steatosis and reduced TG, TC, LDL-C, and oxidative stress in HFD-fed mice and cats by modulating the NF-κB/IκB signaling pathway [16].

Based on traditional medical knowledge and modern pharmaceutical techniques, CB-02 capsules were developed at the Military Medical University using a combination of these three medicinal plants, which were collected from Cao Bang province—an area with favorable soil and climatic conditions for high-quality herbal production. However, the biological efficacy of this preparation in regulating lipid metabolism and controlling BW has not yet been thoroughly evaluated.

Therefore, this study was conducted to evaluate the lipid-modulating and weight-reducing effects of CB-02 capsules in an HFD-induced obesity model using white mice, with the aim of providing scientific evidence for the development of herbal-based therapeutic products derived from Vietnamese medicinal plants.

Although the pharmacological activities of *P. emblica*, *G. pentaphyllum*, and *D. catenatum* have been individually documented, no prior study has systematically investigated their combined formulation in a standardized capsule dosage form. This work represents the first comprehensive evaluation of CB-02, a preparation containing these three medicinal plants, in an HFD-induced obese mouse model. Unlike previous reports, our study assessed a broad spectrum of metabolic parameters, including body weight, adiposity, lipid profiles, insulin sensitivity, and histopathological alterations in both hepatic and pancreatic tissues, while directly benchmarking CB-02 against orlistat, a clinically established anti-obesity agent. Notably, CB-02 not only demonstrated comparable efficacy to orlistat but also provided broader protective effects, underscoring its potential as a multifunctional natural therapeutic. By integrating traditional Vietnamese medicinal plants with modern pharmaceutical preparations, this study provides novel and robust scientific evidence supporting the development of safe, effective, and culturally relevant herbal-based interventions for obesity and related metabolic disorders.

## 2. Results

### 2.1. Obesity Model Induction Results

After 12 weeks of feeding 50 mice an HFD, the BW and BL of all animals were measured. The Lee obesity index was subsequently calculated for each mouse, and 40 mice with a Lee obesity index greater than 310 were selected for inclusion in the main experimental phase. The control group of the main trial consisted of 10 additional healthy, normal male mice weighing 20–25 g. Table 1 below summarizes the key baseline parameters of the mice in all study groups at the beginning of Phase 2.

Mice in the G2, G3, G4, and G5 groups all had Lee obesity indices greater than 310. The BW, AC, and Lee obesity index values in these groups were significantly higher than those of G1 (*p* < 0.001). However, there was no significant difference in BL among the five groups (*p* > 0.05). Obese mice in G2 to G5 showed no significant differences in BW, BL, AC, or the Lee obesity index among each other (*p* > 0.05).

### 2.2. Effects of CB-02 on BW, AC, and the Lee Obesity Index

**Figure 1:**
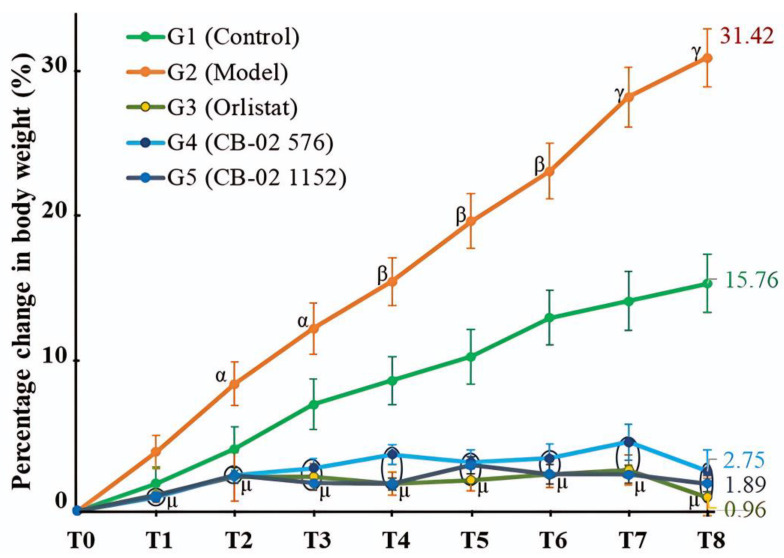
Percentage change in the BW of mice in different groups over time (Mean ± SE). T0: week 0; T1: week 1; T2: week 2; T3: week 3; T4: week 4; T5: week 5; T6: week 6; T7: week 7; and T8: week 8. ^α^ *p* < 0.05; ^β^ *p* < 0.01; ^γ^ *p* < 0.001 compared with G1; ^μ^ *p* < 0.001 compared with G2 (G3, G4, G5 vs. G2).

The BW of mice in both G1 and G2 increased over time. Notably, the weight gain in G2 was significantly greater than that of G1 (*p* < 0.05 at T2 and T3; *p* < 0.01 at T4, T5, and T6; and *p* < 0.001 at T7 and T8). At week 8 (T8), the percentage increase in BW compared to the baseline (T0) in G2 reached 31.42% (Figure 1). In contrast, mice in G3, G4, and G5 exhibited minimal weight gain throughout the 8-week experimental period. Compared to the G2 group, the percentage change in BW from T1 to T8 in all treated groups (G3, G4, and G5) was significantly lower, with *p* < 0.001 at all time points. These results suggest that CB-02, similarly to orlistat, effectively suppressed weight gain in HFD-induced obese mice during the intervention period.

**Figure 2:**
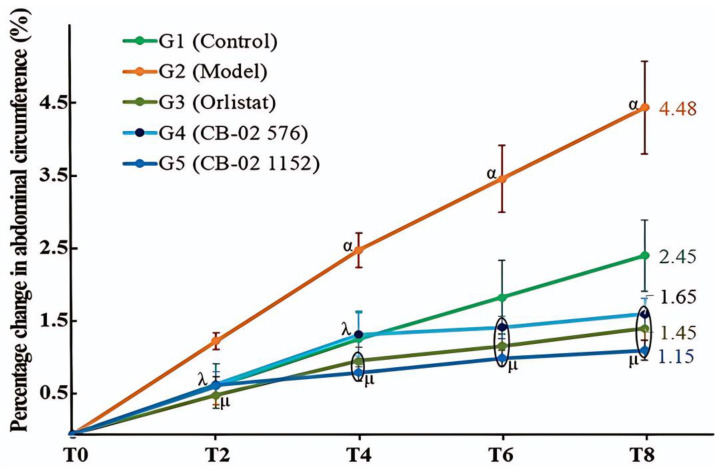
Percentage change in the AC of mice in different groups over time (Mean ± SE). T0: week 0; T2: week 2; T4: week 4; T6: week 6; and T8: week 8. ^α^ *p* < 0.05 compared with G1 (G2 vs. G1); ^λ^ *p* < 0.01 compared with G2; ^μ^ *p* < 0.001 compared with G2 (G3, G4, G5 vs. G2).

AC in both the model (G2) and control (G1) groups increased over time, with a greater increase observed in the model group. At time points T4, T6, and T8, the percentage increase in AC in the model group (G2) was significantly higher than that in the control group (G1, *p* < 0.05) (Figure 2).

By the end of the 8-week experimental period (T8), AC had increased by 4.48% in the model group (G2) and by 2.45% in the control group (G1) compared to the baseline (T0). In comparison with the model group (G2), the groups treated with CB-02 and/or orlistat (G3, G4, and G5) exhibited significantly lower percentage increases in AC (*p* < 0.01 and *p* < 0.001, respectively). At T8, the percentage increased relative to T0 in the CB-02 576 and 1152 mg/kg/day groups, and the orlistat groups were 1.65%, 1.15%, and 1.45% (Figure 2).

**Figure 3:**
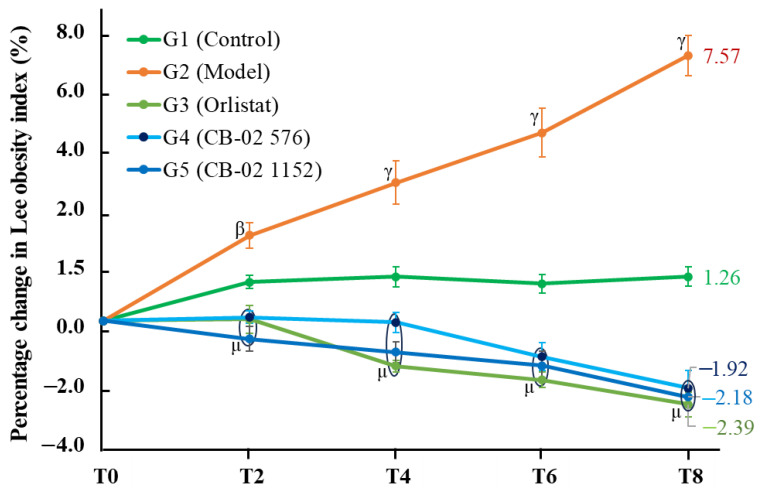
Percentage change in the Lee obesity index of mice in different groups over time (Mean ± SE). T0: week 0; T2: week 2; T4: week 4; T6: week 6; and T8: week 8. ^β^ *p* < 0.01, ^γ^ *p* < 0.001 compared with G1 (G2 vs. G1); ^μ^ *p* < 0.001 compared with G2 (G3, G4, G5 vs. G2).

The Lee obesity index increased over time in both G1 and G2, with a greater increase observed in the latter. Compared to G1, the percentage change in the Lee index in the G2 group was significantly higher, with *p* < 0.01 at time point T2 and *p* < 0.001 at T4, T6, and T8. In contrast, the groups treated with orlistat and CB-02 formulations (G3, G4, and G5) exhibited a downward trend in the Lee obesity index. Compared to the G2 group, the percentage changes in the Lee obesity index from T2 to T8 were significantly lower in all treated groups (*p* < 0.001). At the end of the 8-week intervention (T8), the Lee index increased by 7.57% in the G2 group and 1.26% in the G1 group (Figure 3). In contrast, the Lee index decreased by 2.39% in the orlistat group (G3), 1.92% in the G4 group (576 mg/kg/day), and 2.18% in the G5 group (1152 mg/kg/day).

The values for BW, AC, and the Lee obesity index at the end of the experiment are presented in Table 2.

At the end of the 8-week experimental period, BW, AC, and the Lee obesity index in G2 to G5 were significantly different from those in the control group (G1), with *p* < 0.001. Comparisons between the model group (G2) and the groups treated with orlistat or CB-02 (G3, G4, and G5) revealed statistically significant reductions in BW and the Lee obesity index (*p* < 0.001). Additionally, AC in the orlistat and CB-02 groups (G3, G4, and G5) was significantly lower than that in the model group (G2, *p* < 0.05). No significant differences were observed in BW, AC, or the Lee obesity index among the orlistat- and CB-02-treated groups (*p* > 0.05).

### 2.3. Effects of CB-02 on Organ and Visceral Fat Weights

Compared to the control group (G1), the weights of the heart, liver, kidneys, spleen, and pancreas were significantly increased in the model group (G2, *p* < 0.001). Treatment with either orlistat (G3) or CB-02 (G4 and G5) significantly reduced the weights of these organs compared to the model group (*p* < 0.001); however, the values remained higher than those of the control group (*p* < 0.001 and *p* < 0.01, respectively). No statistically significant differences were observed in the weights of the heart, liver, kidneys, spleen, and pancreas between the orlistat group and the two CB-02 groups (*p* > 0.05) (Table 3).

Compared to the control group (G1), the relative weights of the heart, liver, kidneys, and spleen were significantly increased in the model group (G2, *p* < 0.01). Treatment with orlistat (G3) and CB-02 (G4 and G5) at doses of 576 mg/kg/day and 1152 mg/kg/day significantly reduced the relative weights of the heart, liver, and kidneys compared to the model group (*p* < 0.05), whereas the reduction in the relative weight of the spleen was not statistically significant (*p* > 0.05). The relative weight of the pancreas in the model group (G2) tended to decrease compared to the control group (G1), while treatment with CB-02 (G4 and G5) showed a trend toward increased pancreatic weight relative to the model group (G2); however, these differences were not statistically significant (*p* > 0.05). No statistically significant differences were observed in the relative weights of the heart, liver, kidneys, spleen, and pancreas between the orlistat group (G3) and the two CB-02 groups (G4 and G5, *p* > 0.05) (Table 4).

Compared to the control group (G1), both the absolute and relative weights of visceral adipose tissues in the model group (G2) were significantly increased (*p* < 0.001 and *p* < 0.01, respectively). Treatment with orlistat (G3) or CB-02 (G4 and G5) significantly reduced the absolute and relative weights of these visceral fat depots compared to the model group (G2: *p* < 0.001, *p* < 0.01, and *p* < 0.05). The relative weights of retroperitoneal fat and epididymal fat pads were reduced to levels comparable to those of the control group (G1); however, the relative weight of mesenteric fat remained significantly higher than that in the control group (G2, *p* < 0.01). No statistically significant differences were observed in either the absolute or relative weights of visceral adipose tissues between the orlistat group (G3) and the two CB-02 groups (G4 and G5, *p* > 0.05) (Table 5).

### 2.4. Effects of CB-02 on Blood Lipid Parameters

Compared to G1, G2 showed significantly elevated levels of TC, TG, LDL-C, and non-HDL-C, while HDL-C levels were significantly reduced (*p* < 0.001). Treatment with G3, G4, and G5 significantly improved the lipid profile, as evidenced by reductions in TC, TG, LDL-C, and non-HDL-C levels, and a marked increase in HDL-C levels compared to the model group (*p* < 0.001). However, the levels of TC, TG, LDL-C, and non-HDL-C in G3, G4, and G5 remained significantly higher than those in G1 (*p* < 0.001), while HDL-C levels were restored to values statistically comparable to those of G1 (*p* > 0.05). No statistically significant differences were observed in any of the lipid indices between G3 and either G4 or G5 (*p* > 0.05), indicating comparable efficacy (Table 6).

### 2.5. Effects of CB-02 on Blood Glucose Levels and Insulin Resistance

Compared to the control group (G1), the model group (G2) exhibited significant increases in blood glucose, serum insulin, and HOMA-IR indices, along with significant decreases in the QUICKI and HOMA-β indices (*p* < 0.001). Treatment with orlistat (G3) or CB-02 (G4 and G5) significantly reduced blood glucose and HOMA-IR levels and increased QUICKI and HOMA-β indices compared to the model group (G2, *p* < 0.001), while serum insulin levels were also significantly decreased (*p* < 0.01). However, blood glucose, serum insulin, HOMA-IR, and QUICKI indices did not return to levels comparable to those of the control group (G1). The HOMA-β index in the orlistat-treated group (G3) was restored to a level comparable to the control group (G1), whereas in the CB-02-treated groups (G4 and G5) the HOMA-β index was significantly higher than that of the control group (G1, *p* < 0.05 and *p* < 0.01, respectively). Compared to the orlistat group (G3), both CB-02 groups (G4 and G5) showed significantly lower blood glucose levels and significantly higher HOMA-β indices (*p* < 0.01). No statistically significant differences were observed between the two CB-02 groups (G4 and G5) in blood glucose, serum insulin, or insulin resistance indices (*p* > 0.05) (Table 7).

### 2.6. Histopathological Images of the Pancreas and Liver from Mice in Different Experimental Groups

**Figure 4:**
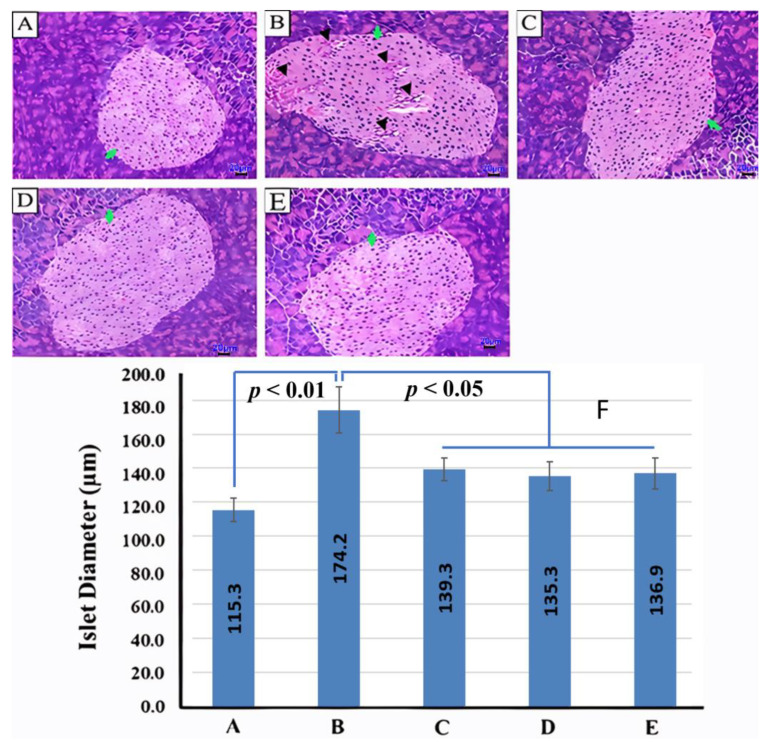
Histopathological features of H&E-stained pancreatic tissue and the islet diameter of Langerhans. (**A**): Control group (G1); (**B**): Model group (G2); (**C**): Orlistat group (G3); (**D**): CB-02 576 group (G4); (**E**): CB-02 1152 group (G5); (**F**): Islet diameter of Langerhans (Mean ± SE). Green arrows: the islets of Langerhans; black arrows: cellular morphological changes and disorganized tissue architecture. Scale bar: 20 µm.

Pancreatic lobules were separated by thin fibrous septa. The exocrine acini contained abundant eosinophilic secretions. The islets of Langerhans (indicated by green arrows) consisted of cells with lightly stained cytoplasm. In the model group (G2), the islets were markedly enlarged compared to those in the control group (G1), with some regions exhibiting cellular morphological changes and disorganized tissue architecture (black arrows). In the orlistat- and CB-02-treated groups (G3, G4, and G5), the islet size was reduced relative to the model group (G2), and only minimal cellular and structural abnormalities were observed (Figure 4A–E). The islet diameter in the model group (G2) was significantly greater than that in the control group (G1, *p* < 0.01). In contrast, treatment with orlistat or CB-02 significantly decreased islet diameter compared to the model group (*p* < 0.05). No statistically significant differences were observed in islet diameter among the three treatment groups (orlistat and CB-02) (Figure 4F).

**Figure 5:**
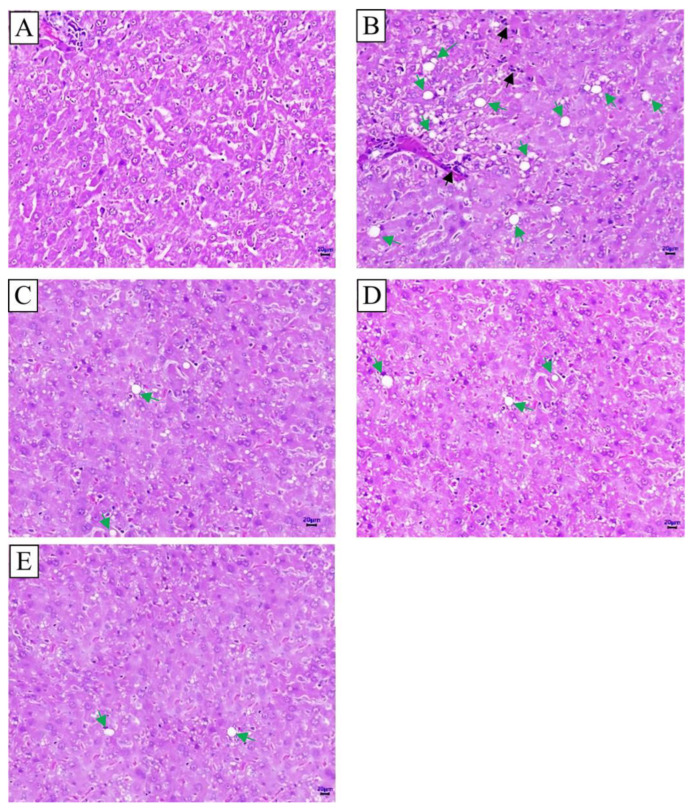
Histopathological features of liver tissue stained with H&E. (**A**): Control group (G1); (**B**): Model group (G2); (**C**): Orlistat group (G3); (**D**): CB-02 576 group (G4); (**E**): CB-02 1152 group (G5). Green arrows indicate lipid droplets within the hepatic parenchyma, and black arrows indicate infiltration of inflammatory cells. Scale bar: 20 µm.

In the model group (G2), the prominent accumulation of lipid droplets was observed within the hepatic parenchyma (green arrows), along with the notable infiltration of inflammatory cells (black arrows). In contrast, the orlistat- and CB-02-treated groups (G3, G4, and G5) exhibited a marked reduction in hepatic lipid accumulation and minimal to no inflammatory cell infiltration (Figure 5). No significant histopathological differences were detected among the three treatment groups (orlistat and both CB-02 doses).

## 3. Discussion

In experimental research evaluating anti-obesity effects, the establishment and utilization of animal models of obesity play a critical role in recapitulating the pathophysiological processes of the disease and providing a reliable basis for objectively assessing the efficacy of therapeutic interventions. Various approaches have been developed to induce obesity in animals, including hypercaloric feeding, the administration of high-energy or HFDs, hypothalamic damage (via surgical or chemical means), and the use of genetically modified strains with mutations affecting energy metabolism [17,18,19]. Among these, the HFD-induced obesity model is the most employed one due to its high feasibility, reproducible outcomes, and versatility for different research purposes. HFD regimens typically provide 45% to 60% of their total energy from fat and are administered to rodents for durations ranging from 8 to 27 weeks, depending on the experimental design and modeling intensity [20,21,22].

In this study, an HFD-induced obesity model was employed, in which the diet provided a total energy content of 4495.8 kcal/kg, with fat accounting for 48.08% of the total caloric intake. The administration of an HFD increases caloric consumption, disrupts metabolic balance, and promotes lipid accumulation in adipose tissue and various organs, thereby inducing obesity and associated metabolic dysfunctions. Within a few weeks of HFD exposure, experimental animals typically develop key features resembling human metabolic syndrome, including rapid weight gain, hyperglycemia, dyslipidemia, hypertension, insulin resistance, and endothelial dysfunction—all of which are major risk factors for cardiovascular diseases and type 2 diabetes [19,22].

Notably, mice are one of the most widely used models in obesity research due to their genetic homogeneity, high susceptibility to HFD-induced obesity, and sensitivity to metabolic perturbations. Mice are also advantageous for laboratory use because of their small size, low maintenance cost, ease of handling, and rapid physiological responses—making them an ideal choice for preliminary investigations into pathophysiological mechanisms and therapeutic interventions. Moreover, the HFD-induced obesity model in mice allows for the comprehensive evaluation of biochemical parameters (e.g., serum lipids, blood glucose), organ and fat pad weights, and the expression of genes and proteins involved in energy metabolism and inflammatory pathways. The degree of obesity and metabolic derangement in mice can be flexibly modulated by adjusting the dietary fat content, feeding duration, and mouse strain used. Therefore, this model not only closely mimics the pathophysiological characteristics of human obesity but also serves as a robust platform for assessing the efficacy of pharmacological agents, herbal medicines, nutraceuticals, or lifestyle interventions.

This study was conducted in two distinct phases. During Phase 1, which lasted 12 weeks, mice were fed an HFD to induce obesity. Phase 2 consisted of an 8-week intervention period, during which the obese mice continued receiving the HFD and were randomly assigned to treatment groups administered either the test formulation or the reference drug, orlistat. Anti-obesity efficacy was evaluated based on a set of predefined physiological and metabolic parameters. Orlistat was chosen as the positive control due to its established clinical use as a Food and Drug Administration-approved anti-obesity medication since 1999, and its official adoption in Vietnam since 2008. Orlistat is a hydrogenated derivative of lipstatin—a natural inhibitor of pancreatic lipase originally isolated from *Streptomyces toxytricini*. It exerts its therapeutic effect by selectively inhibiting gastrointestinal lipases, particularly pancreatic lipase, thereby preventing the hydrolysis of dietary triglycerides into absorbable FFA and monoglycerides. This results in reduced intestinal fat absorption. Notably, orlistat does not interfere with other digestive enzymes such as trypsin, chymotrypsin, amylase, or various esterases, and therefore does not impair the digestion or absorption of carbohydrates or proteins [23].

To preliminarily assess obesity in mice, several basic morphometric indices were employed, including BW, BL (measured from the tip of the nose to the anus), AC, and the Lee obesity index. Among these, the Lee obesity index is a commonly used parameter in preclinical studies to reflect fat distribution and the degree of obesity in rodents. Analogous to the body mass index in humans, the Lee obesity index is calculated based on BW and BL. Additionally, AC serves as a valuable complementary indicator, reflecting visceral fat accumulation, which is closely associated with metabolic disorders, including insulin resistance, dyslipidemia, and metabolic syndrome. The combination of multiple morphometric parameters enhances the accuracy and objectivity of obesity assessment in animal models. Our findings indicated that, following 12 weeks of HFD feeding, all mice in G2 through to G5 exhibited Lee obesity index values exceeding the threshold of 310, commonly accepted as the cut-off for obesity in rodents. The mean Lee obesity index values in these groups (G2–G5) were all above 326 and were significantly higher than those in the control group (G1, *p* < 0.001) (Table 1).

Furthermore, the HFD induced a marked increase in both BW and AC in mice compared to the control group (G1, *p* < 0.001), indicating substantial fat accumulation during the induction period. In contrast, the average BL (measured from the nose to the anus) of mice in the HFD-fed groups showed only a slight, statistically non-significant increase relative to controls (*p* > 0.05). This can be explained by the fact that the animals used were adults, whose linear growth had stabilized; therefore, changes primarily occurred in terms of body mass and fat distribution. These results collectively confirm that all the mice included in the intervention phase had developed a clearly defined obese phenotype, meeting the criteria for inclusion in the subsequent treatment experiment.

In experimental obesity research, an increase in BW is commonly regarded as a clear and quantifiable indicator of excessive fat accumulation. Mariana et al. [22] noted that, in most diet-induced obesity models using energy-dense feeding, BW gain is the principal parameter employed to evaluate the onset and progression of obesity. Consequently, controlling and reducing BW is considered a critical criterion for assessing the therapeutic efficacy of anti-obesity interventions. An analysis of the percentage change in BW over the 8-week intervention period revealed that the control group (G1), maintained on a standard chow diet, exhibited normal physiological growth with an average increase of 15.76%. In contrast, the model group (G2), which continued to receive an HFD, showed a marked weight gain averaging 31.42%, indicating a sustained and pronounced trend of adiposity induced by the obesogenic diet.

The HFD formulation used in this study contained a high fat content (48.08%), which has the potential to activate central regulatory systems by increasing the expression of NPY—a neurotransmitter that stimulates feeding behavior. Elevated NPY levels not only promote hunger and increase food intake but also reduce energy expenditure by suppressing thermogenesis, thereby leading to enhanced fat accumulation [24]. This mechanism explains the observation of the rapid weight gain in the group of mice that continued to consume the HFD. Although the mice were continuously fed the HFD, those in the orlistat-treated and CB-02-treated groups (G3, G4, and G5) showed minimal weight gain, and even slight weight loss was observed in the orlistat group (G3, BW change at week 8: –0.96%). Orlistat acts by inhibiting pancreatic lipase, thus blocking the hydrolysis of dietary fats into monoglycerides and FFA [23,25], which leads to a reduction in fat absorption of approximately 30% [26]. Consequently, reduced fat absorption decreases calorie intake and contributes to weight loss. CB-02 exhibited a weight-reducing effect comparable to that of orlistat (Table 2). Although the exact mechanism of CB-02 has not been fully elucidated, based on the known pharmacological actions of its herbal components, CB-02 is thought to exert its anti-obesity effects through multiple targets. *P. emblica* has been shown to promote weight loss via AMPK activation, the inhibition of adipogenic gene expression, modulation of the gut microbiota, and reduction of hepatic lipid synthesis [11,27,28]. *G. pentaphyllum* has demonstrated anti-obesity effects through the activation of the AMPK–SIRT1 pathway, the enhancement of lipoprotein lipase activity, the promotion of fatty acid oxidation, and the inhibition of lipogenesis [13,14,29]. *D. catenatum* has been reported to reduce BW by inhibiting lipogenesis, enhancing β-oxidation, regulating the microbiota–insulin axis, and reducing inflammation [16,30,31]. The therapeutic targets of CB-02 are likely to involve multiple pathways derived from its constituent herbs, warranting further investigation to clarify its underlying mechanisms.

Similarly to BW, changes in AC and the Lee obesity index were also selected as key parameters with which to evaluate the anti-obesity effects of CB-02. These parameters were measured after 2, 4, 6, and 8 weeks of CB-02 administration. As shown in Figure 2 and Figure 3, the model group (G2) exhibited a marked and progressive increase in both AC and the Lee obesity index over time, with statistically significant percentage changes compared to the control group (G1). The model group (G2) was maintained on an HFD, which promotes lipogenesis while suppressing lipid oxidation. Excess dietary fat that is not metabolized is stored in adipocytes, particularly in the abdominal region, resulting in an increase in abdominal girth and the Lee obesity index. In contrast, mice treated with CB-02 (G4 and G5) or orlistat (G3) showed significantly lower increases in both AC and the Lee obesity index compared to the model group (G2, *p* < 0.01 and *p* < 0.001, respectively). The reductions in these indices, alongside BW loss, serve as fundamental criteria supporting the anti-obesity efficacy of CB-02. These effects are presumably mediated through mechanisms similar to those involved in the observed weight reduction, as discussed above.

Organ and adipose tissue weight indices are critical biological parameters in obesity research, particularly in animal models. Evaluating these indices provides valuable insights into pathophysiology, metabolism, and therapeutic efficacy. Adipose tissue weight directly reflects excessive energy accumulation, a hallmark of obesity. Notably, visceral fat is strongly associated with insulin resistance, dyslipidemia, and chronic inflammation. A reduction in adipose tissue mass, especially visceral fat, following treatment is considered a key indicator of the biological efficacy of anti-obesity drugs or herbal preparations [32,33].

Relative organ weight, defined as the percentage of organ weight relative to total BW, serves as an objective quantitative index with which to diagnose the degree of obesity in animals, assess treatment effectiveness, and monitor potential side effects on vital organs such as the liver, kidneys, and heart. An increase in relative liver weight in obesity is primarily attributed to hepatic fat accumulation (hepatic steatosis), along with multiple pathogenic mechanisms involving lipid metabolism disorders, insulin resistance, and chronic inflammation [34]. The elevation of relative heart weight is a commonly observed phenomenon in both experimental and clinical studies of obesity. This increase is driven by several pathophysiological factors, including mechanical overload due to increased body mass, obesity-induced hypertension, and chronic low-grade inflammation. The latter involves elevated levels of inflammatory cytokines such as TNF-α, IL-6, and leptin, which can directly damage cardiac tissue, leading to myocardial fibrosis and cardiomyocyte hypertrophy, thus increasing heart weight [35]. An increase in relative kidney weight is often linked to glomerular hyperfiltration and albuminuria, associated with insulin resistance and metabolic syndrome. Renal hypertrophy in obesity is a well-documented pathophysiological phenomenon, particularly in animal models and obese individuals. This renal enlargement reflects multiple pathological changes, including glomerular hyperfiltration, interstitial hyperplasia, and lipid accumulation within the kidneys [36]. Splenomegaly, or increased relative spleen weight, is also observed in numerous experimental studies, especially in HFD-induced obese animal models. Although the spleen is not a central metabolic organ like the liver or kidneys, increases in spleen weight reflect obesity-related immune dysregulation, chronic inflammation, and enhanced immune activity [37]. Our study showed that the relative weights of visceral adipose tissues and organs in the model group (G2) were significantly elevated compared to the control group (G1), reflecting the characteristic pathological alterations of obesity in the experimental model. The administration of CB-02 at doses of 576 and 1152 mg/kg/day significantly reduced the relative weights of visceral fat depots, and the liver, heart, and kidneys compared to the model group (G2), with effects comparable to those of orlistat at 60 mg/kg/day (Table 3 and Table 4), thereby confirming the anti-obesity efficacy of CB-02. The relative spleen weight showed a decreasing trend after CB-02 and orlistat administration (G3, G4, and G5), though it was not statistically significant, possibly due to the longer time required to reverse obesity-induced splenic alterations.

Regarding the pancreas, the absolute pancreatic weight in the model group (G2) increased significantly compared to the control group (G1, *p* < 0.001), accompanied by a significant enlargement of the diameter of the pancreatic islets (*p* < 0.01). Compared with the model group (G2), both the orlistat- and CB-02-treated groups (G3, G4, and G5) showed a marked reduction in absolute pancreatic weight (*p* < 0.001), as well as a decrease in islet diameter (*p* < 0.05). However, no statistically significant differences were observed in terms of relative pancreatic weight (i.e., pancreas weight normalized to BW) between the model and control groups (G2 and G1), or between the treatment groups (G3, G4, and G5) and the model group (G2, *p* > 0.05). These results suggest that changes in absolute pancreatic weight and islet diameter may correspond to changes in overall BW. In obesity, endocrine pancreatic tissue—particularly the islets of Langerhans—may undergo the partial hypertrophy of β-cells; however, such changes are typically insufficient to cause a notable increase in total pancreatic mass, while exocrine pancreatic tissue remains largely unaffected. Prolonged obesity, characterized by persistent elevations in circulating lipids and glucose, may also induce lipotoxic and glucotoxic stress on β-cells, leading to impaired function and the gradual loss of β-cell mass, ultimately resulting in mild pancreatic atrophy over time [38].

An HFD induces dyslipidemia through multiple interrelated mechanisms, including the enhanced absorption of exogenous lipids, the stimulation of hepatic lipid synthesis, insulin resistance, reduced activity of lipolytic enzymes, and chronic inflammation. These pathophysiological changes result in elevated levels of TG and LDL-C, and reduced HDL-C [39]. The present findings demonstrated that both orlistat and CB-02 significantly reduced serum levels of TC, TG, LDL-C, and non-HDL-C, while increasing HDL-C levels compared to the model group (*p* < 0.001) (Table 6). Orlistat acts as a pancreatic lipase inhibitor, impeding the enzymatic breakdown of dietary fats in the gastrointestinal tract, thereby reducing lipid absorption. CB-02, a polyherbal formulation containing *P. emblica*, *G. pentaphyllum*, and *D. catenatum*, may exert its therapeutic effects through a multi-target approach, resulting from the complementary pharmacological actions of its constituent herbs.

*P. emblica* has been shown to activate AMPK, inhibit lipogenesis by downregulating PPARγ, SREBP-1c, ACC, and FAS, and enhance lipid β-oxidation through the upregulation of CPT-1 and PPARα [11]. Saponins and flavonoids derived from *G. pentaphyllum* similarly activate AMPK, thereby reducing the expression of SREBP-1c, ACC, and FAS, while upregulating PGC-1α, UCP-1, and CPT-1 to promote mitochondrial fatty acid oxidation. Additionally, this herb regulates cholesterol homeostasis via the modulation of SREBP-2, 3-hydroxy-3-methylglutaryl-CoA reductase, and LDL receptor expression, and enhances bile acid synthesis through the farnesoid X receptor/cholesterol 7α-hydroxylase signaling axis [40]. *D. catenatum* activates the PPAR-retinoid X receptor and AMPK signaling pathways to increase fatty acid oxidation, regulate hepatic metabolomic profiles, and reduce lipotoxicity [38]. Furthermore, it exhibits pronounced anti-inflammatory effects by inhibiting the NF-κB/IκB pathway, mitigates oxidative stress (as evidenced by decreased malondialdehyde and reactive oxygen species levels), and improves lipid profiles, including TG, TC, and the LDL-C/HDL-C ratio [14]. Collectively, the herbal components of CB-02 demonstrate potent bioactivity in regulating lipid metabolism and are likely to act synergistically on the three major pathological pathways of dyslipidemia: (1) inhibition of endogenous lipid synthesis via the downregulation of transcriptional regulators such as SREBP-1c, ACC, FAS, and PPARγ, resulting in a reduction in the hepatic production of triglycerides and cholesterol; (2) promotion of lipid catabolism and β-oxidation through AMPK activation and the upregulation of PPARα, CPT-1, UCP-1, and PGC-1α [11,40]; and (3) attenuation of lipid absorption as well as enhancement of lipid excretion [40,41]. In addition, all three botanical ingredients in CB-02 possess strong antioxidant properties, contributing to anti-inflammatory effects, hepatoprotection, and improved systemic metabolic function [12,13,16]. The synergistic integration of these medicinal herbs enables CB-02 to exert a comprehensive therapeutic effect on dyslipidemia, particularly that induced by high-fat dietary intake.

An HFD induces hyperglycemia primarily by reducing insulin sensitivity in hepatic, adipose, and muscle tissues, leading to decreased glucose uptake and increased hepatic gluconeogenesis. As blood glucose is not efficiently transported into cells, the pancreas compensates by secreting more insulin, resulting in hyperinsulinemia [42]. To assess the degree of insulin resistance and pancreatic β-cell function, QUICKI, HOMA-IR, and HOMA-β were evaluated [43]. QUICKI reflects overall insulin sensitivity, while HOMA-IR indicates the extent of insulin resistance. A decrease in QUICKI and an increase in HOMA-IR are indicative of insulin resistance. The HOMA-β index estimates the insulin secretory function of pancreatic β-cells; a higher value suggests stronger β-cell activity.

As shown in Table 7, the model group (G2) exhibited increased HOMA-IR and decreased QUICKI, indicating that an HFD led to significant insulin resistance, subsequently resulting in elevated blood glucose and compensatory hyperinsulinemia. The HOMA-β index in the model group was also reduced (Table 7), suggesting a decline in pancreatic compensatory function. Although insulin secretion remained high, it was insufficient to maintain glucose homeostasis, resulting in marked hyperglycemia. Treatment with orlistat and CB-02 significantly lowered blood glucose levels, increased QUICKI, reduced HOMA-IR, and elevated HOMA-β (Table 7). Notably, CB-02 exhibited a stronger effect in reducing blood glucose and increasing HOMA-β compared to orlistat (*p* < 0.01). Furthermore, both CB-02-treated groups (G4 and G5) displayed significantly higher HOMA-β values than the control group (G1, *p* < 0.05 and *p* < 0.01, respectively). This suggests that CB-02 may exert its glucose-lowering effects through multiple mechanisms beyond those of orlistat. In obese mice, orlistat reduces lipid absorption, which improves insulin resistance, lowers inflammation, and restores pancreatic β-cell function. Similarly, CB-02 improved dyslipidemia and thereby reduced insulin resistance. Additionally, CB-02 may exert its antihyperglycemic effects through synergistic mechanisms involving its herbal components: *G. pentaphyllum*, *D. catenatum*, and *P. emblica*.

The ethyl acetate extract from *P. emblica* L. (EPE) has been shown to enhance AMPK phosphorylation in liver and skeletal muscle tissues and to increase glucose transporter type 4 expression on muscle cell membranes. EPE treatment also suppressed hepatic gluconeogenesis by downregulating G6Pase and PEPCK, and reduced glycogen synthase kinase 3β phosphorylation, thereby modulating hepatic glycogen synthesis, contributing to antidiabetic and insulin-sensitizing effects [28]. The ethanol extract from *P. emblica* fruit further reduced the intestinal absorption of sucrose and glucose [44].

*G. pentaphyllum*, rich in gypenosides and polysaccharides, lowers blood glucose through mechanisms such as enhancing glucokinase activity, inhibiting hepatic glucose-producing enzymes like G6Pase, promoting pancreatic β-cell insulin secretion, improving insulin signaling, reducing insulin resistance, and suppressing glucose absorption [29,45,46]. Clinical studies have confirmed that Vietnamese *G. pentaphyllum* tea possesses antidiabetic effects through enhancing insulin sensitivity [47].

Polysaccharides isolated from the stems and leaves of *D. catenatum* have demonstrated pronounced hypoglycemic activity in type 2 diabetic mouse models. These polysaccharides promote hepatic glycogen synthesis and inhibit glycogen breakdown by suppressing the glucagon–cAMP–PKA signaling pathway. Simultaneously, they activate the PI3K/Akt pathway, increasing glycogen synthesis and enhancing glucose-metabolizing enzyme activities [48]. Additionally, compounds such as bibenzyls and phenanthrenes isolated from *D. catenatum* have shown potent α-glucosidase inhibitory activity, with N-*p*-coumaroyltyramine exhibiting an IC_50_ of approximately 0.4 µM, significantly more potent than acarbose [49].

CB-02, a formulation composed of *P. emblica*, *G. pentaphyllum*, and *D. catenatum*, may exert its antihyperglycemic effects via multiple mechanisms. All three herbs enhance glucose uptake by activating AMPK or PI3K/Akt pathways, promote glucose transport into cells, and stimulate glycogen synthesis. They also inhibit intestinal glucose absorption, thereby reducing postprandial blood glucose, and possess antioxidant, anti-inflammatory, and lipid-lowering properties that help protect pancreatic β-cells and improve insulin sensitivity. Furthermore, CB-02 may increase glucose utilization by upregulating glucose-metabolizing enzymes (e.g., hexokinase, glucose-6-phosphate dehydrogenase), suppress gluconeogenesis by inhibiting PEPCK and G6Pase, and reduce glycogenolysis by inhibiting glucagon-mediated cAMP–PKA signaling. These potential mechanisms warrant further investigation in future studies.

In obesity, particularly that induced by an HFD, the most evident pathological changes are observed in the liver and pancreas, accompanied by disturbances in lipid and carbohydrate metabolism. Therefore, histological examinations of these organs were conducted to evaluate the therapeutic effects of the tested formulation. In the model group (G2), liver sections revealed clear signs of steatosis and inflammatory cell infiltration. Pancreatic histology showed the hypertrophy of the islets of Langerhans, with certain areas exhibiting morphological alterations and disorganization of tissue architecture. The degeneration of pancreatic β-cells was also observed, with these cells being replaced by inflammatory cells and fibrotic tissue. Treatment with the reference drug orlistat (G3) and the CB-02 formulation (G4 and G5) significantly attenuated these pathological features in both the liver and pancreas of obese mice (Figure 4 and Figure 5). These results suggest that CB-02 provides protective effects to both hepatic and pancreatic tissues under metabolic stress. The underlying mechanisms are likely related to its antioxidant and anti-inflammatory properties, as well as its ability to regulate lipid and glucose metabolism, thereby protecting hepatocytes and pancreatic β-cells from oxidative stress and chronic inflammation and improving their functional integrity. These findings further support the therapeutic potential of CB-02 in managing type 2 diabetes, dyslipidemia, and non-alcoholic fatty liver disease.

The findings of this study demonstrated that an HFD successfully induced obesity in experimental animals, as evidenced by significant increases in body weight, adipose tissue mass, and indicators of insulin resistance. These results align with previous studies confirming that HFD models effectively replicate the pathophysiological mechanisms of obesity, including excessive lipid accumulation, chronic low-grade inflammation, and metabolic disturbances [50,51]. Such models are widely utilized in obesity research to simulate human conditions and provide a reliable basis for evaluating potential therapeutic interventions [50].

The administration of CB-02 extract significantly reduced body weight and fat accumulation compared with the HFD group, showing comparable or even superior effects to orlistat. The weight-reducing effect of CB-02 may be associated with the synergistic action of its constituent herbs, including *P. emblica*, *G. pentaphyllum*, and *D. catenatum*. Previous research has demonstrated that *P. emblica* contains phenolic and flavonoid compounds with potent antioxidant activity, which can regulate lipid metabolism and mitigate oxidative stress [52,53]. Similarly, *G. pentaphyllum* has been reported to modulate energy metabolism through AMPK activation, leading to decreased lipogenesis and enhanced fatty acid oxidation [54]. In addition, *D. catenatum* contributes polysaccharides with hypoglycemic and lipid-lowering properties [16,55].

These findings suggest that CB-02 may act through multiple metabolic pathways to control obesity, in contrast to orlistat, which primarily functions by inhibiting gastrointestinal lipase. Furthermore, CB-02 treatment improved glycemic control and reduced insulin resistance in obese mice, as evidenced by decreased fasting blood glucose, lower HOMA-IR indices (Table 7), and improved glucose tolerance. These outcomes are consistent with earlier studies on herbal formulations rich in polyphenols and saponins, which exert anti-diabetic effects by enhancing insulin sensitivity and protecting pancreatic β-cells [53,54]. Specifically, gypenosides from *G. pentaphyllum* have been shown to improve glucose uptake and regulate insulin signaling pathways [54]. This mechanism differs from orlistat, which has a limited impact on insulin sensitivity despite its weight-reducing effect.

At the molecular level, obesity is characterized by excessive release of free fatty acids, which promotes lipotoxicity, oxidative stress, and the activation of pro-inflammatory cytokines such as TNF-α and IL-6, contributing to insulin resistance [51]. Polyphenols and polysaccharides present in CB-02 may alleviate these processes by reducing oxidative stress and inflammation, thereby improving insulin action. This explanation is supported by previous studies demonstrating that *P. emblica* polyphenols inhibit lipid peroxidation and protect against oxidative stress–induced damage [52], while *D. catenatum* polysaccharides modulate gut microbiota and enhance glucose homeostasis [55,56].

Taken together, the results indicate that CB-02 not only reduces body weight but also exerts beneficial effects on glucose metabolism and insulin sensitivity through multi-target mechanisms involving antioxidant, anti-inflammatory, and metabolic regulatory activities. This multifaceted action distinguishes CB-02 from orlistat, suggesting its potential as a safe and effective alternative or complementary therapeutic strategy for obesity and related metabolic disorders.

## 4. Materials and Methods

### 4.1. CB-02 Capsules

CB-02 hard capsules meeting institutional quality standards were manufactured by Phu Tin Pharmaceutical Joint Stock Company (Thuong Tin, Hanoi, Vietnam). Each capsule contains 400 mg of CB-02 dry extract powder, corresponding to 224 mg of dry extract of *P. emblica*, 86 mg of dry extract of *D. catenatum*, and 90 mg of dry extract of *G. pentaphyllum*. CB-02 capsules were formulated and evaluated according to quality standards, including quantification of key active constituents, with each capsule containing not less than 11.0 mg of gallic acid, 0.27 mg of ginsenoside Rb1, and 60.0 mg of total polysaccharides (Supplementary Materials). The capsules were stored in sealed brown PET bottles with aluminum sealing membranes and silica gel desiccant, protected from light. Before administration, the capsules were opened, and the powder was suspended in distilled water to form a dense suspension (1 g of powder per 1.5 mL of distilled water) for oral gavage in experimental animals. The preparation process of dried extracts from *G. pentaphyllum*, *D. catenatum*, and *P. emblica* is described as follows:

#### 4.1.1. Preparation of *G. pentaphyllum* Dried Extract

Raw material of *G. pentaphyllum* that meets quality standards was ground into coarse powder and subjected to ultrasonic extraction at 60 MHz, with extraction temperature set at 60 °C, solvent 80% ethanol, extraction duration 60 min per cycle, and repeated twice at a solvent-to-material ratio of 6:1. The extract was filtered and concentrated into a 1:1 liquid extract (equivalent to 1 g of crude material per 1 mL of liquid extract) under reduced pressure (300 mmHg) at 60 °C. The dried extract was subsequently obtained by spray drying under the following conditions: inlet temperature 140 ± 2 °C, excipients aerosil:maltodextrin (60:40), excipient-to-solid ratio 20%, solid concentration in the feed solution 15%, feed rate 30 mL/min, and pump pressure 0.2 MPa.

#### 4.1.2. Preparation of *D. catenatum* Dried Extract

Raw *D. catenatum* meeting quality standards was ground into coarse powder and extracted ultrasonically at 60 MHz, with extraction temperature 70 °C, solvent water, extraction time 90 min per cycle, and repeated twice at a solvent-to-material ratio of 8:1. The filtrate was concentrated into a 1:1 liquid extract (1 g of crude material per 1 mL of liquid extract) under reduced pressure (300 mmHg) at 60 °C. The dried extract was then obtained by spray drying under the following conditions: inlet temperature 140 ± 2 °C, excipients aerosil:maltodextrin (80:20), excipient-to-solid ratio 30%, solid concentration in the feed solution 25%, feed rate 30 mL/min, and pump pressure 0.2 MPa.

#### 4.1.3. Preparation of *P. emblica* Dried Extract

Dried *P. emblica* fruit that satisfied quality standards was crushed and extracted with 50% ethanol at boiling temperature, with an extraction time of 90 min per cycle and repeated twice at a solvent-to-material ratio of 4:1. The extract was filtered and concentrated under reduced pressure (300 mmHg) at 60 °C. The concentrated extract was then vacuum-dried at a pressure of 10 mmHg and a drying temperature of 60 °C to obtain the dried extract (moisture content < 5%).

### 4.2. Animals

Adult, healthy male Swiss albino mice, weighing 16–18 g, were obtained from the Laboratory Animal Center of the Military Medical University. Mice were housed under standard laboratory conditions, fed a standard diet, and provided with water ad libitum.

### 4.3. Equipment and Reagents

An analytical balance (CP224S) and a technical balance were used, both manufactured by Sartorius (Göttingen, Germany), and biochemical analyses were performed using a biochemical analyzer from Biochemical Systems International Srl (Arezzo, Italy). Enzyme-linked immunosorbent assays (ELISAs) were conducted using a 96-well plate reader (Thermo, Vantaa, Finland), with a refrigerated centrifuge (Universal 320) used for sample processing (Hettich, Tuttlingen, Germany). Blood glucose levels were measured using test strips and the OneTouch Profile Meter (Johnson & Johnson, New Brunswick, NJ, USA). Commercial assay kits were employed for the quantification of TC, TG, and HDL-C, and capsules of 10 mg of orlistat were provided by STADA (Ho Chi Minh City, Vietnam).

### 4.4. In Vivo Test

#### 4.4.1. Phase 1—Induction of Obesity

Male Swiss albino mice (14–16 g, 5–6 weeks old) were acclimatized for one week under controlled conditions (22 ± 2 °C, 12 h light/dark cycle, 50–60% humidity) with ad libitum access to food and water. Obesity was induced by feeding the animals an HFD for 12 consecutive weeks [51]. The relative caloric contribution of macronutrients in the HFD is presented in Table 8.

After 12 weeks, obesity in mice was assessed using the Lee obesity index [57], calculated using Formula (1):

(1) $$Lee\;obesity\;index=\frac{\sqrt[{3}]{BW}}{BL}\times 1000$$

where BW (g) and (BL) from nose to anus (cm). Mice with a Lee obesity index ≥ 310.0 were classified as obese [58]. A total of 40 obese mice were selected for Phase 2 of the study.

#### 4.4.2. Phase 2—Main Experiment

A total of 40 obese Swiss albino mice obtained from Phase 1 and 10 additional healthy male Swiss albino mice (20–25 g) were included in Phase 2. The animals were randomly allocated into five groups (*n* = 10 per group) and maintained on their assigned diets and treatment regimens for 8 weeks as follows:

+Group 1 (G1, Control): Healthy mice fed a standard diet and administered distilled water (10 mL/kg).

+Group 2 (G2, Model): Obese mice maintained on HFD and given distilled water (10 mL/kg).

+Group 3 (G3, Orlistat): Obese mice maintained on HFD and treated with orlistat (60 mg/kg/day).

+Group 4 (G4, CB-02 576): Obese mice maintained on HFD and treated with CB-02 suspension (576 mg/kg/day).

+Group 5 (G5, CB-02 1152): Obese mice maintained on HFD and treated with CB-02 suspension (1152 mg/kg/day).

#### 4.4.3. Anthropometric and Obesity-Related Parameters

The BW was recorded weekly throughout the 8-week intervention. Body length (BL) was measured under light anesthesia (1% propofol, 0.1 mL/10 g, i.p.) from the tip of the nose to the anus with 0.1 cm precision. Waist circumference was measured immediately after BL at identical time points. Assessments were performed at baseline (T0), and at weeks 2, 4, 6, and 8 (T2–T8) [59]. The Lee obesity index was calculated biweekly according to Formula (1) [57].

#### 4.4.4. Biochemical Measurements

At the end of the experiment, mice were fasted overnight before blood collection from the orbital sinus. Serum lipid parameters (TC, TG, HDL-C) were quantified using commercial assay kits. Blood glucose levels were determined using glucose strips and a OneTouch Profile Meter, while serum insulin concentrations were measured by ELISA. LDL-C and non-HDL-C were derived using the Friedewald equations (Formulas (2) and (3)) [60]:

(2) $$LDL-C=TC-HDL-C-\frac{TG}{2.2}\;\;$$

(3) Non HDL−C=TC−HDL−C

#### 4.4.5. Insulin Resistance Indices

The indices QUICKI, HOMA-IR, and HOMA-β were calculated using standard formulas [43].

$$QUICKI=\frac{1}{log\;(fasting\;plasma\;glucose\;[mg/dL])+log\;(fasting\;plasma\;insulin\;[µIU/mL])}$$

$$HOMA-IR=\frac{fasting\;plasma\;glucose\;(mg/dL)+log\;fasting\;plasma\;insulin\;(µIU/mL)}{405}$$

$$HOMA-\beta =\frac{360\times fasting\;plasma\;insulin\;(µIU/mL)}{fasting\;plasma\;glucose\;(mg/dL)}$$

#### 4.4.6. Organ and Fat Tissue Analysis

Following sacrifice, major organs (liver, spleen, kidneys, heart, pancreas) were excised, rinsed in 0.9% cold saline, blotted, and weighed. Relative organ weight was calculated according to Formula (4). Visceral fat pads (mesenteric, retroperitoneal, epididymal) were dissected, cleaned, weighed, and expressed as relative fat weight using Formula (5).

(4) $$Relative\;organ\;weight=\frac{Organ\;weight}{Body\;weight}\times 100\;(\%)$$

(5) $$Relative\;fat\;weight=\frac{Fat\;weight}{Body\;weight}\times 100\;(\%)$$

#### 4.4.7. Histopathology

Histopathological slides of the pancreas and liver were stained with H&E and examined at the Department of Pathological Anatomy and Forensic Medicine, Military Hospital 103. ImageJ software (version 1.53e, National Institutes of Health, Bethesda, MD, USA) was used to measure the diameter of the islets of Langerhans in the mouse pancreas.

### 4.5. Statistical Analysis

All data are expressed as mean ± standard error (SE). Normality and homogeneity of variance were tested prior to analysis. Comparisons among groups were performed using one-way analysis of variance (ANOVA), followed by Tukey’s post hoc test for pairwise comparisons. A *p*-value < 0.05 was considered statistically significant. Analyses were conducted using SPSS version 22.0.

## 5. Conclusions

This study demonstrates that the oral administration of CB-02 at 576 and 1152 mg/kg/day significantly improves obesity-related parameters, dyslipidemia, and hyperglycemia in HFD-induced obese Swiss albino mice. CB-02 reduced BW gain, AC, and the Lee obesity index compared to the model group, with effects comparable to orlistat (60 mg/kg/day) and independent of dose. Visceral fat depots (retroperitoneal, epididymal, and mesenteric) and relative weights of the liver, heart, and kidneys also decreased significantly. Regarding lipid metabolism, CB-02 lowered TC, TG, LDL-C, and non-HDL-C, while increasing HDL-C. CB-02 also reduced hepatic steatosis and inflammatory cell infiltration in liver histopathological sections, with effects similar to those of orlistat and no significant dose-dependent differences. CB-02 improved glycemic control by lowering fasting blood glucose, increasing QUICKI, reducing HOMA-IR, and enhancing HOMA-β, indicating improved insulin sensitivity and β-cell function. These effects were more pronounced than those with orlistat. Moreover, CB-02 exhibited protective effects on pancreatic β-cells by attenuating morphological alterations and histopathological changes in pancreatic tissue. Overall, CB-02 shows potential as a multifunctional therapeutic candidate for obesity and its metabolic complications, including dyslipidemia, hyperglycemia, hepatic steatosis, and insulin resistance, thereby contributing to the prevention of type 2 diabetes and cardiovascular disease.

## Figures and Tables

**Table 1:** Baseline parameters of mice at the beginning of the main experimental phase.

Group	*n*	Research Indicators at T0 (Mean ± SE)			
		BW (g)	BL (cm)	AC (mm)	Lee Obesity Index
G1 (1)	10	22.02 ± 0.66	9.69 ± 0.10	69.50 ± 0.36	289.05 ± 1.70
G2 (2)	10	33.93 ± 1.53 ^γ^	9.89 ± 0.11	81.35 ± 0.84 ^γ^	326.65 ± 2.54 ^γ^
G3 (3)	10	34.38 ± 1.42 ^γ^	9.85 ± 0.12	81.72 ± 0.85 ^γ^	329.58 ± 2.01 ^γ^
G4 (4)	10	32.67 ± 1.13 ^γ^	9.79 ± 0.10	82.02 ± 0.35 ^γ^	326.17 ± 0.98 ^γ^
G5 (5)	10	33.06 ± 1.41 ^γ^	9.82 ± 0.11	81.84 ± 0.38 ^γ^	326.25 ± 2.00 ^γ^

The *p*-values were determined using one-way ANOVA followed by Tukey’s post hoc test to compare BW, BL, AC, and the Lee obesity index among the experimental groups at the baseline of the formal trial. ^γ^ indicates *p* < 0.001 compared with G1.

**Table 2:** BW, AC, and the Lee obesity index at the end of the main experimental phase.

Group	*n*	Research Indicators at T8 (Mean ± SE)		
		BW (g)	AC (mm)	Lee Obesity Index
G1 (1)	10	25.79 ± 0.70	71.19 ± 0.21	292.66 ± 1.45
G2 (2)	10	44.49 ± 1.81 ^γ,μ^	84.96 ± 0.56 ^γ,κ^	351.36 ± 3.20 ^γ,μ^
G3 (3)	10	34.58 ± 1.10 ^γ,μ^	82.90 ± 0.81 ^γ,κ^	321.70 ± 2.20 ^γ,μ^
G4 (4)	10	33.58 ± 1.35 ^γ,μ^	83.37 ± 0.39 ^γ,κ^	319.89 ± 1.83 ^γ,μ^
G5 (5)	10	33.67 ± 1.40 ^γ,μ^	82.78 ± 0.41 ^γ,κ^	319.12 ± 1.71 ^γ,μ^

The *p*-values were calculated using one-way ANOVA followed by Tukey’s post hoc test to assess differences in BW, AC, and the Lee obesity index among the study groups at the end of the experimental period. ^γ^ indicates *p* < 0.001 compared with G1; ^κ^ indicates *p* < 0.05 and ^μ^ indicates *p* < 0.001 compared with G2.

**Table 3:** Organ weights of mice across the experimental groups.

Group	*n*	Organ Weights (g) (Mean ± SE)				
		Heart	Liver	Kidneys	Spleen	Pancreas
G1 (1)	10	0.125 ± 0.006	1.119 ± 0.059	0.241 ± 0.008	0.083 ± 0.004	0.228 ± 0.009
G2 (2)	10	0.255 ± 0.008 ^γ^	2.495 ± 0.072 ^γ^	0.521 ± 0.017 ^γ^	0.193 ± 0.010 ^γ^	0.349 ± 0.011 ^γ^
G3 (3)	10	0.171 ± 0.006 ^γ,μ^	1.647 ± 0.082 ^γ,μ^	0.343 ± 0.015 ^γ,μ^	0.119 ± 0.010 ^β,μ^	0.276 ± 0.012 ^β,μ^
G4 (4)	10	0.170 ± 0.007 ^γ,μ^	1.626 ± 0.045 ^γ,μ^	0.348 ± 0.016 ^γ,μ^	0.123 ± 0.009 ^β,μ^	0.281 ± 0.013 ^β,μ^
G5 (5)	10	0.163 ± 0.007 ^γ,μ^	1.584 ± 0.075 ^γ,μ^	0.339 ± 0.018 ^γ,μ^	0.116 ± 0.008 ^β,μ^	0.286 ± 0.011 ^β,μ^

The *p*-values were determined using one-way ANOVA followed by Tukey’s post hoc test to compare absolute organ weights among the experimental groups. ^β^ and ^γ^ indicate *p* < 0.01 and *p* < 0.001, respectively, compared with G1; ^μ^ indicates *p* < 0.001 compared with G2.

**Table 4:** Relative organ weights of mice to BW across the experimental groups.

Group	*n*	Relative Organ Weight to BW (%) (Mean ± SE)				
		Heart	Liver	Kidneys	Spleen	Pancreas
G1 (1)	10	0.484 ± 0.022	4.339 ± 0.197	0.943 ± 0.046	0.323 ± 0.020	0.885 ± 0.033
G2 (2)	10	0.579 ± 0.023 ^β^	5.712 ± 0.317 ^β^	1.192 ± 0.067 ^β^	0.445 ± 0.036 ^β^	0.792 ± 0.032 ^β^
G3 (3)	10	0.498 ± 0.021 ^κ^	4.803 ± 0.273 ^κ^	1.002 ± 0.054 ^κ^	0.346 ± 0.031	0.799 ± 0.030
G4 (4)	10	0.510 ± 0.018 ^κ^	4.888 ± 0.195 ^κ^	1.036 ± 0.028 ^κ^	0.365 ± 0.021	0.840 ± 0.031
G5 (5)	10	0.491 ± 0.029 ^κ^	4.755 ± 0.250 ^κ^	1.013 ± 0.046 ^κ^	0.350 ± 0.028	0.855 ± 0.037

The *p*-values were determined using one-way ANOVA followed by Tukey’s post hoc test to compare relative organ weights among the experimental groups. ^β^ indicates *p* < 0.01 compared with G1; ^κ^ indicates *p* < 0.05 compared with G2.

**Table 5:** Weights of visceral fat in mice across the experimental groups (Mean ± SE).

Group	*n*	Visceral Fat Weight (mg)			Relative Visceral Fat Weight to BW (%)		
		Mesenteric Artery Fat	Retroperito-Neal Fat	Epididymal Fat	Mesenteric Artery Fat	Retroperitoneal Fat	Epididymal Fat
G1 (1)	10	228.38 ± 14.67	192.29 ± 17.42	424.69 ± 22.20	0.890 ± 0.059	0.749 ± 0.066	1.650 ± 0.084
G2 (2)	10	766.22 ± 26.33 ^γ^	510.06 ± 29.17 ^γ^	1050.26 ± 33.04 ^γ^	1.743 ± 0.082 ^γ^	1.165 ± 0.083 ^β^	2.403 ± 0.135 ^γ^
G3 (3)	10	410.44 ± 22.05 ^μ,γ^	297.84 ± 25.08 ^μ,β^	619.53 ± 30.59 ^μ,γ^	1.189 ± 0.056 ^μ,β^	0.876 ± 0.090 ^κ^	1.818 ± 0.120 ^λ^
G4 (4)	10	426.88 ± 25.88 ^μ,γ^	309.01 ± 30.64 ^μ,β^	629.56 ± 26.29 ^μ,γ^	1.278 ± 0.076 ^μ,β^	0.914 ± 0.079 ^κ^	1.885 ± 0.007 ^λ^
G5 (5)	10	393.38 ± 24.28 ^μ,γ^	287.31 ± 25.88 ^μ,β^	616.11 ± 31.43 ^μ,γ^	1.175 ± 0.070 ^μ,β^	0.863 ± 0.086 ^κ^	1.853 ± 0.106 ^λ^

The *p*-values were determined using one-way ANOVA followed by Tukey’s post hoc test to compare absolute and relative weights of visceral fat tissues among the experimental groups. ^β^, ^γ^ indicate *p* < 0.01 and *p* < 0.001 compared with G1, respectively; ^κ^, ^λ^, ^μ^ indicate *p* < 0.05, *p* < 0.01, and *p* < 0.001 compared with G2, respectively.

**Table 6:** Blood lipid profiles of mice in experimental groups (Mean ± SE).

Group	*n*	Blood Lipid Indices (mmol/L)				
		TC	TG	HDL-C	LDL-C	Non-HDL-C
G1 (1)	10	3.22 ± 0.16	0.76 ± 0.04	1.58 ± 0.06	1.29 ± 0.18	1.63 ± 0.19
G2 (2)	10	6.46 ± 0.27 ^γ^	1.79 ± 0.10 ^γ^	1.01 ± 0.05 ^γ^	4.63 ± 0.21 ^γ^	5.45 ± 0.26 ^β^
G3 (3)	10	4.45 ± 0.27 ^μ,γ^	1.12 ± 0.06 ^μ,β^	1.46 ± 0.07 ^μ,γ^	2.49 ± 0.25 ^μ,β^	2.99 ± 0.27 ^μ^
G4 (4)	10	4.85 ± 0.22 ^μ,γ^	1.27 ± 0.06 ^μ,β^	1.39 ± 0.08 ^μ,γ^	2.88 ± 0.22 ^μ,β^	3.46 ± 0.25 ^μ^
G5 (5)	10	4.54 ± 0.20 ^μ,γ^	1.16 ± 0.05 ^μ,β^	1.49 ± 0.07 ^μ,γ^	2.52 ± 0.22 ^μ,β^	3.05 ± 0.24 ^μ^

The *p*-values were calculated using one-way ANOVA followed by Tukey’s post hoc test to compare blood lipid indices among the experimental groups. ^β^, ^γ^ indicate *p* < 0.01 and *p* < 0.001 compared with G1, respectively; ^μ^ indicates *p* < 0.001 compared with G2.

**Table 7:** Blood glucose, serum insulin, and insulin resistance indices in mice (Mean ± SE).

Group	*n*	Blood Glucose (mg/dL)	Serum Insulin (µIU/mL)	Insulin Resistance Indices		
				QUICKI	HOMA-IR	HOMA-β
G1 (1)	10	96.40 ± 2.95	3.22 ± 0.21	0.404 ± 0.006	0.77 ± 0.07	35.91 ± 2.46
G2 (2)	10	179.34 ± 7.80 ^γ^	7.54 ± 0.30 ^γ^	0.320 ± 0.003 ^γ^	3.35 ± 0.20 ^γ^	24.14 ± 1.54 ^γ^
G3 (3)	10	127.76 ± 2.69 ^μ,γ^	6.19 ± 0.27 ^λ,γ^	0.346 ± 0.003 ^μ,γ^	1.96 ± 0.12 ^μ,γ^	34.64 ± 1.35 ^μ^
G4 (4)	10	115.18 ± 3.35 ^μ,β,ε^	6.23 ± 0.19 ^λ,γ^	0.351 ± 0.003 ^μ,γ^	1.77 ± 0.08 ^μ,γ^	44.29 ± 2.65 ^μ,α,ε^
G5 (5)	10	111.06 ± 4.96 ^μ,α,ε^	6.28 ± 0.28 ^λ,γ^	0.353 ± 0.004 ^μ,γ^	1.74 ± 0.13 ^μ,γ^	50.81 ± 4.40 ^μ,β,ε^

The *p*-values were calculated using one-way ANOVA followed by Tukey’s post hoc test to compare blood glucose, serum insulin, and insulin resistance indices among the experimental groups. ^α^, ^β^, ^γ^ indicate *p* < 0.05, *p* < 0.01, and *p* < 0.001 compared with G1, respectively; ^λ,^,^μ^ indicate *p* < 0.01 and *p* < 0.001 compared with G2, respectively; ^ε^ indicates *p* < 0.01 compared with G3.

**Table 8:** The relative caloric contribution of macronutrients in the HFD and standard diet.

Nutrient	HFD		Standard Diet	
	Energy (Kcal)	Percentage (%)	Energy (Kcal)	Percentage (%)
Protein	641.0	14.26	520.6	17.02
Carbohydrate	1693.4	37.67	2173.1	71.04
Fat	2161.4	48.08	365.2	11.94
Total	4495.8	100	3058.9	100

## Data Availability

The original contributions presented in this study are included in the article. Further inquiries can be directed to the corresponding authors.

## Supplementary Materials

The following supporting information can be downloaded at: https://www.mdpi.com/article/10.3390/molecules30183678/s1, Figure S1: Chromatograms of blank sample (A), gallic acid standard solution (B), and sample (C); Figure S2: Chromatograms of the blank sample (A), the ginsenoside Rb1 standard solution (B), and the test sample (C); Figure S3: Standard calibration curve depicting the relationship between the concentration of the glucose standard solution and the optical density; Table S1: Validation results of the quantitative analysis method for gallic acid; Table S2: Quantification results of gallic acid content in CB-02 capsules; Table S3: Validation results of the quantitative analytical method for ginsenoside Rb1; Table S4: Quantification results of GRb1 in hard capsules; Table S5: Optical density of glucose standard solutions at different concentrations; and Table S6: Total polysaccharide content in capsules.

## Abbreviations

The following abbreviations are used in this manuscript:

AC	Abdominal circumference
ACC	Acetyl-CoA carboxylase
Akt	Protein kinase B
AMPKα	AMP-activated protein kinase subunit alpha
BL	Body length
BW	Body weight
cAMP–PKA	Cyclic adenosine monophosphate-protein kinase A
CPT-1	Carnitine palmitoyltransferase 1
FAS	Fatty acid synthase
FFA	Free fatty acid
G6Pase	Glucose-6-phosphatase
H&E	Hematoxylin and eosin
HDL-C	High-density lipoprotein cholesterol (HDL-C)
HFD	High-fat diet
HOMA-IR	Homeostatic model assessment for insulin resistance
HOMA-β	Homeostatic model assessment for β-cell function
HSL	Hormone-sensitive lipase
IL-6	Interleukin-6
LDL-C	Low-density lipoprotein cholesterol
NF-κB/IκB	Nuclear factor kappa-light-chain-enhancer of activated B-cells/inhibitor of kappa light polypeptide gene enhancer in B-cells
Non-HDL-C	Non-high-density lipoprotein cholesterol
NPY	Neuropeptide Y
PEPCK	Phosphoenolpyruvate carboxykinase
PGC-1α	Peroxisome proliferator-activated receptor gamma coactivator 1-alpha
PI3K	Phosphoinositide 3-kinase
PPARα	Peroxisome proliferator activated receptor alpha
QUICKI	Quantitative insulin sensitivity check index
SIRT1	Sirtuin 1
SREBP-1c	Sterol regulatory element-binding protein 1c
TC	Total cholesterol
TG	Triglyceride
TNF-α	Tumor necrosis factor-alpha
UCP-1	Uncoupling protein 1
