# Supplementary material for: Anti-Obesity, Lipid-Lowering, and Anti-Hyperglycemic Effects of CB-02 in High-Fat-Diet-Induced Obese Mice
Source: Molecules. 2025 Sep 10;30(18):3678. doi: 10.3390/molecules30183678 (PMC12472232; doi:10.3390/molecules30183678)
Supplement: Supplementary file 1 [file molecules-30-03678-s001.zip › molecules-3828344-supplementary.pdf]

Anti-Obesity, Lipid-Lowering, and Anti-Hyperglycemic Effects of CB-02 in High-Fat-Diet-Induced Obese Mice

Hoang Lan Hiep [1], Phung Van Bang [1], Nguyen Dinh Nhan [1], Nguyen Hoang Ngan [2], Dao Cuong To [3], Nguyen Van Dung [4], and Le Hong Phu [1,*]

[1] Military Institute of Traditional Medicine, 442 Kim Giang, Dinh Cong, Hanoi 11717, Vietnam; hoanghiep.yhct@gmail.com; phungbangbs8@gmail.com; nhantom@yahoo.com; hongphu70@yahoo.com

[2] Vietnam Military Medical University, 160 Phung Hung, Ha Dong, Hanoi 12108, Vietnam; nganvmu@gmail.com

[3] Phenikaa University Nano Institute (PHENA), Phenikaa School of Engineering (PSE), Phenikaa University, Nguyen Van Trac, Duong Noi, Hanoi 12116, Vietnam; cuong.todao@phenikaa-uni.edu.vn

[4] Thai Nguyen University of Medicine and Pharmacy, 284 Luong Ngoc Quyen, Phan Dinh Phung, Thai Nguyen 24124, Vietnam; dung.dhdtn@gmail.com

* Correspondence: hongphu70@yahoo.com; Tel.: +84 985735088

1. Quantification of Gallic Acid by HPLC

The analysis was performed according to the method described in the Chinese Pharmacopoeia (2015), *Phyllanthi Fructus* monograph.

1.1. Sample Preparation

Standard solution: Accurately weigh approximately 10.0 mg of gallic acid and dissolve it in a 10 mL volumetric flask containing 50% methanol to obtain a stock standard solution. Serial dilutions of the stock solution were prepared with 50% methanol to yield working standard solutions at appropriate concentrations.

Test solution: Accurately weigh an amount of powdered tablet equivalent to the average mass of one tablet (480 mg) and transfer it into a conical flask. Add approximately 150 mL of 50% methanol, sonicate until completely dissolved, and transfer the solution into a 200 mL volumetric flask. Dilute to volume with 50% methanol. Subsequently, pipette 1 mL of this solution into a 10 mL volumetric flask and dilute to volume with 50% methanol to obtain the test solution for chromatography.

Blank solution: 50% methanol was used as the blank solution for sample preparation. All solutions were filtered through a 0.45 µm membrane filter prior to chromatographic analysis.

1.2. Chromatographic Conditions

Chromatographic separation was performed on a C18 column (5 µm, 4.5 × 250 mm). The column oven was maintained at 40 °C with a flow rate of 1.0 mL/min. The injection volume was 10 µL, and the detection wavelength was set at 273 nm. The mobile phase consisted of methanol and 0.2% phosphoric acid, applied under the following gradient elution program:

**Time**	**Methanol (%)**	**Phosphoric acid 0.2% (%)**
0-10	5	95
10-14	5-90	95-10
14-25	90	10
25-29	90-5	10-95
29-35	5	95

1.3. Establishment of the Standard Curve

Standard solutions with concentrations ranging from 5 to 75 µg/mL (specifically, 5, 10, 20, 25, 50, and 75 µg/mL) were prepared by serial dilution of the initial stock standard solution using appropriate dilution factors. Each standard solution was injected three times into the chromatographic system. Chromatograms were recorded, and the peak areas were determined.

A calibration curve was constructed by plotting the peak area (y) against the concentration of gallic acid (x, µg/mL). The linear regression equation and the correlation coefficient (R^2) were calculated using the least-squares method to evaluate the linear relationship between concentration and peak response.

1.4. Quantification of Test Samples

The test solutions were injected individually, each in triplicate. Chromatograms were recorded, and the peak areas corresponding to gallic acid were measured.

The concentration of gallic acid in the test solution was calculated according to the following formula:

$$C\ (\mu g/mL) = \frac{S - b}{a} \times \frac{H}{100}$$

where

S = peak area of gallic acid (µV·s),

a, b = regression coefficients of the calibration curve,

H = purity of the reference substance (%).

The gallic acid content in the capsule was determined using the following formula:

$$X\ (mg/capsule) = \frac{C \text{ x } 10 \times 200 \times M}{1000 \times m} \times \frac{100}{100 - a}$$

where

X = gallic acid content (mg/capsule),

C = concentration of gallic acid in the test solution (µg/mL),

m = mass of the drug powder used for analysis (mg),

M = average mass of the capsule (mg),

a = moisture content of the drug powder (%).

2. Quantification of Ginsenoside Rb1 by HPLC

2.1. Sample Preparation

Standard solution: Approximately 5.0 mg of ginsenoside Rb1 reference standard was accurately weighed and dissolved in a 5 mL volumetric flask with methanol to obtain a stock standard solution (1 mg/mL). The stock solution was serially diluted with methanol to prepare working standard solutions at appropriate concentrations.

Test solution: An amount of powdered tablet equivalent to the average mass of one tablet (480 mg) was accurately weighed and transferred into a 25 mL volumetric flask. About 20 mL of methanol was added, and the mixture was sonicated for 30 minutes. After cooling, the solution was diluted to volume with methanol and mixed thoroughly.

All solutions were filtered through a 0.45 µm membrane filter prior to HPLC analysis.

2.2. Chromatographic Conditions

Chromatographic separation was performed on a C18 column (250 × 4.6 mm, 5 µm). The column oven temperature was maintained at 25 °C, with a flow rate of 0.8 mL/min. The injection volume was 10 µL, and detection was carried out at 203 nm. The mobile phase consisted of a mixture of 0.01% phosphoric acid and acetonitrile (66:34, v/v).

2.3. Construction of the Standard Curve

Standard solutions with concentrations ranging from 5 to 160 µg/mL (specifically, 5, 10, 20, 40, 80, and 160 µg/mL) were prepared by appropriate dilutions of the stock standard solution. Each solution was injected in triplicate into the chromatographic system. Chromatograms were recorded, and the peak areas were measured.

A calibration curve was constructed by plotting the peak area (y) against the ginsenoside Rb1 concentration (x, µg/mL). The linear regression equation and the coefficient of determination (R^2) were obtained to evaluate the linearity of the calibration curve.

2.4. Quantification of Test Samples

The test solutions were injected individually, each in triplicate. Chromatograms were recorded, and the peak areas corresponding to ginsenoside Rb1were measured.

The concentration of ginsenoside Rb1in the test solution was calculated according to the following formula:

$$C\ (\mu g/mL) = \frac{S - b}{a} \times \frac{H}{100}$$

where

S = peak area of ginsenoside Rb1 (µV·s),

a, b = regression coefficients of the calibration curve,

H = purity of the reference substance (%).

The ginsenoside Rb1 content in the capsule was determined using the following formula:

$$X\ (mg/capsule) = \frac{C \text{ x } 10 \times 200 \times M}{1000 \times m} \times \frac{100}{100 - a}$$

where

X = ginsenoside Rb1 content (mg/capsule),

C = concentration of gallic acid in the test solution (µg/mL),

m = mass of the drug powder used for analysis (mg),

M = average mass of the capsule (mg),

a = moisture content of the drug powder (%).

3. Quantification of Total Polysaccharides by Photometric Method

3.1. Sample Preparation

3.1.1. Standard solution

A glucose stock solution (100 µg/mL) was prepared. From this stock, a series of standard solutions with concentrations ranging from 10 to 60 µg/mL were obtained by appropriate dilution.

The glucose–sulfuric acid–phenol reaction was performed as follows: 1 mL of each standard solution was transferred into a test tube, followed by the addition of 1 mL of 5% phenol solution. Subsequently, 5 mL of concentrated sulfuric acid was added. The mixture was shaken thoroughly and heated in a water bath at 90

°C for 15 minutes to complete the hydrolysis reaction. After heating, the solution was rapidly cooled for 10 minutes to stabilize the temperature, yielding the spectrophotometric solution of the standard sample.

3.1.2. Blank solution

The blank solution was prepared in the same way as the standard solution, except that distilled water was used instead of glucose solution.

3.1.3. Test solution

Approximately 200 mg of capsule powder was accurately weighed and transferred into a 50 mL volumetric flask. Thirty-five milliliters of distilled water was added, and the mixture was sonicated at 70 °C for 30 minutes. After cooling, the solution was diluted to volume with distilled water and mixed thoroughly.

The extract was preliminarily filtered. A 10 mL aliquot of the filtrate was further centrifuged or filtered through filter paper. One milliliter of the clear supernatant was transferred to a 50 mL volumetric flask, diluted to volume with distilled water, mixed well, and filtered through a 0.45 µm membrane filter. Finally, 1 mL of this filtrate was transferred to a test tube and subjected to the same hydrolysis reaction as described for the standard solutions, yielding the spectrophotometric solution of the test sample.

3.2. Construction of the Standard Curve

The optical density (OD) of the standard solutions was measured at 488 nm. A calibration curve was constructed by plotting the OD values against the glucose concentrations (µg/mL).

3.3. Sample Quantification

The OD of the test sample solutions was measured at 488 nm. The concentration of glucose equivalent in the sample photometric solution was calculated according to the following equation:

$$C\ (\mu g/mL) = \frac{Y - b}{a} \times \frac{H}{100}$$

where

Y = optical absorbance of the test solution at 488 nm,

a, b = regression coefficients of the calibration curve,

H = purity of the glucose reference standard (%).

The total polysaccharide content in the capsule, expressed as glucose equivalent, was determined using the following formula:

$$X\ (mg/capsule) = \frac{C \times 50 \times 50 \times M}{1000 \times m} \times \frac{100}{100 - a}$$

where

X = total polysaccharide content (mg/capsule, calculated as glucose),

C = concentration of the test solution (µg/mL),

m = powder mass used for analysis (mg),

M = average mass of the capsule (mg),

a = moisture content of the capsule powder (%).

4. Quantitative Results

4.1. Quantitative Determination of Gallic Acid in Capsules by HPLC

4.1.1. Validation of the Quantitative Analysis Method for Gallic Acid

Table S1. Validation results of the quantitative analysis method for gallic acid

**Indicator**	**Results**
**System suitability**	Peak area (µV·s): S = 796.292; RSD = 0.42%
	Retention time (min): t_R = 9.901; RSD = 0.35%
	Symmetry factor: 1.25
	Number of theoretical plates: 13.576
**Specificity**	Match coefficient > 0.99
**Linearity (n = 6)**	Linear within the range of 1–50 µg/mL. Calibration equation: y = 402.688x – 590.138; correlation coefficient R^2 = 0.9987
**Repeatability (n = 6)**	HL_{tb} = 5.469%; RSD = 1.56%
**Accuracy (n = 6)**	Recovery: 99.55–102.82% (mean = 100.91%); RSD = 1.38%

**LOD**	0.05 µg/mL
**LOQ**	0.165 µg/mL

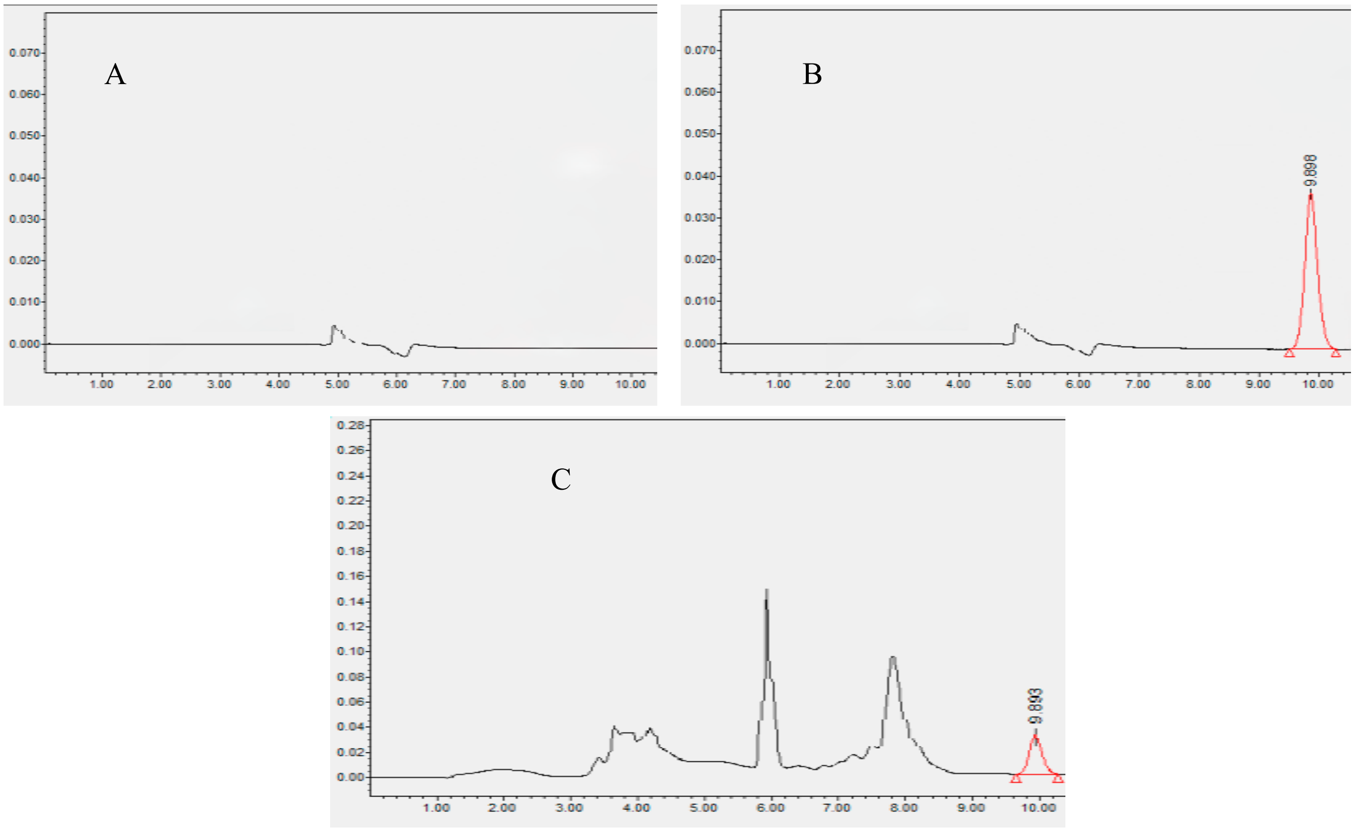

Figure S1. Chromatograms of blank sample (A), gallic acid standard solution (B), and sample (C).

4.1.2. Quantification of Gallic Acid in CB-02 Capsules

Table S2. Quantification results of gallic acid content in CB-02 capsules

**Sample**	**Gallic acid peak area**	**Gallic acid content per capsule (mg)**
1	381272	11.59
2	523495	13.28
3	428684	12.12
4	390161	11.68
5	476083	12.70
**Average ± SD**		**12.28 ± 0.72**

The gallic acid content in CB-02 capsules was determined to be **12.28 ± 0.72 mg/capsule**, ranging from 11.59 to 13.28 mg/capsule. Based on these results, we propose that the quality standard for CB-02 capsules should specify a gallic acid content of not less than 11.0 mg per capsule.

4.2. Quantification of ginsenoside Rb1 in capsules by HPLC

4.2.1. Validation results of the quantitative analytical method for ginsenoside Rb1

Table S3. Validation results of the quantitative analytical method for ginsenoside Rb1

**Indicator**	**Results**
**System compatibility**	Peak area (µV·s): S = 768877; RSD = 0.75
	Retention time (min): t_R = 24.079; RSD = 0.12
	Asymmetry coefficient: 1.12
	Number of theoretical plates: 9489
**Specificity**	Match coefficient > 0.99

**Linear range (n = 5)**	In the concentration range of 5–160 µg/mL, peak area and concentration showed a linear correlation according to the equation y = 87594x + 68042, with a correlation coefficient R^2 = 0.9964
**Repeatability (n = 6)**	HLtb = 0.141%; RSD = 2.35%
**Accuracy (n = 6)**	Recovery rate from 96.68% to 102.12% (average 99.47%); RSD = 1.81%
**LOD**	0.03 µg/mL
**LOQ**	0.10 µg/mL

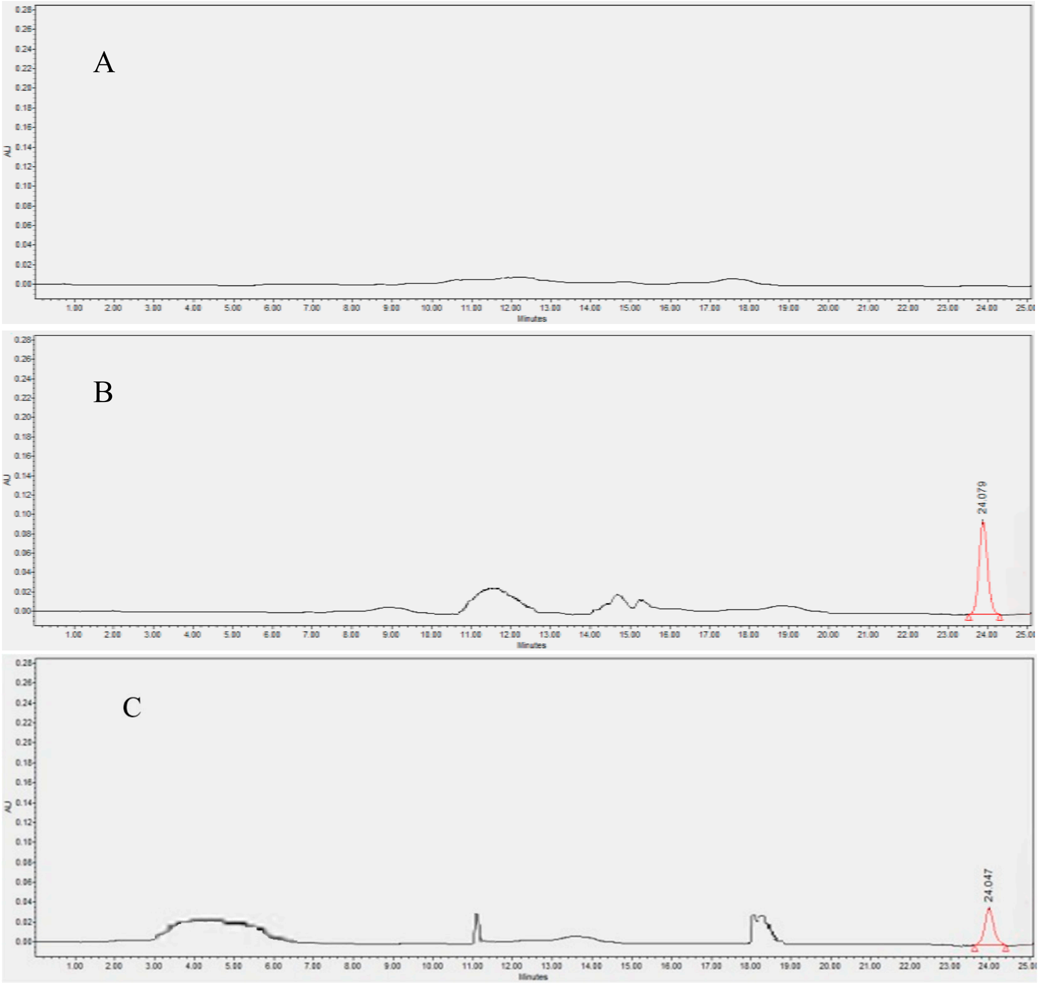

Figure S2. Chromatograms of the blank sample (A), the ginsenoside Rb1 standard solution (B), and the test sample (C).

4.2.2. GRb1 quantification results in CB-02 capsules

Table S4. Quantification results of GRb1 in hard capsules

**Sample**	**GRb1 peak area**	**GRb1 peak area**
1	226771	0.3524
2	181638	0.2965
3	188179	0.3021
4	216687	0.3414
5	205917	0.3269
**Average ± SD**		**0.3239 ± 0.0243**

C

The GRb1 content in the hard capsules was 0.3239 ± 0.0243 mg/capsule, with values ranging from 0.2965 to 0.3524 mg/capsule across the tested samples. Based on these results, we propose that the specification for GRb1 content in CB-02 hard capsules should be not less than 0.27 mg/capsule.

4.3. Quantification of total polysaccharides in capsules by photometric method

4.3.1. Results of glucose standard curve construction

Table S5. Optical density of glucose standard solutions at different concentrations.

Concentration (µg/mL)	Optical density
10	0.1372
20	0.2209
30	0.3311
40	0.4210
50	0.5102
60	0.6119

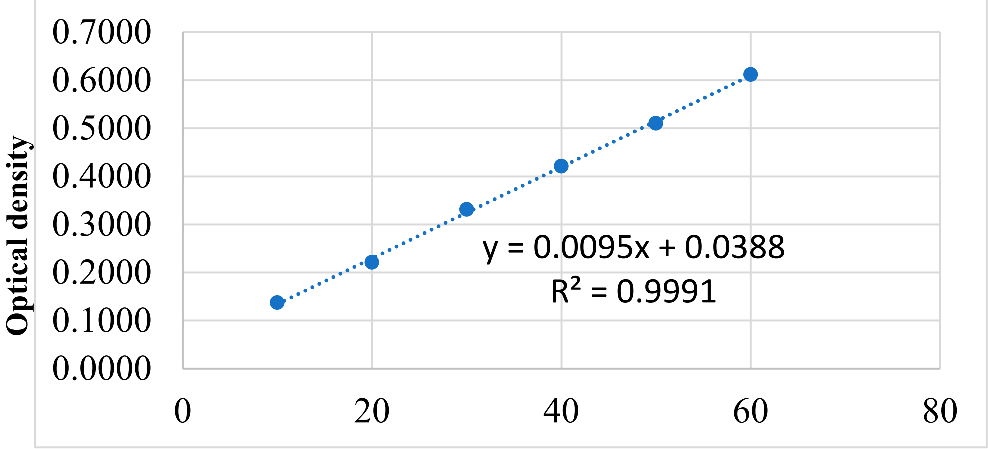

Figure S3. Standard calibration curve depicting the relationship between the concentration of the glucose standard solution and the optical density.

From the results above, it can be observed that the optical density exhibits a linear dependence on glucose concentration, with a correlation coefficient of R^2 = 0.9991. The standard calibration curve is expressed by the equation y = 0.0095x + 0.0388. Therefore, during quantification, the test samples were diluted to ensure that their optical density values fell within the linear range of the calibration curve.

4.3.2. Results of quantification of total polysaccharides in capsules

Table S6. Total polysaccharide content in capsules

Sample	Optical density	Total polysaccharides content per capsule (mg)
1	0.1402	66.08
2	0.1369	63.93
3	0.1372	64.09
4	0.1394	65.56
5	0.1384	64.88
**Average ± SD**		**64.91 ± 0.93**

The results indicate that the total polysaccharide content in hard capsules is 64.91 ± 0.93 mg per capsule. The measured values ranged from 63.93 to 66.08 mg/capsule across the tested samples. Based on these findings, we propose that the quality standard for total polysaccharide content in hard capsules should be set at not less than 60.00 mg per capsule.
